# Occurrence of Natural Interspecific Hybrids in the Pufferfish, Genus *Takifugu*, and Their Tetrodotoxin-accumulating Tissues

**DOI:** 10.14252/foodsafetyfscj.D-25-00021

**Published:** 2026-04-20

**Authors:** Ryohei Tatsuno, Hiroshi Takahashi

**Affiliations:** 1 Department of Food Science and Technology, National Fisheries University, Japan Fisheries Research and Education Agency, 2-7-1 Nagata-Honmachi, Shimonoseki, Yamaguchi 759-6595, Japan; 2 Department of Applied Aquabiology, National Fisheries University, Japan Fisheries Research and Education Agency, 2-7-1 Nagata-Honmachi, Shimonoseki, Yamaguchi 759-6595, Japan

**Keywords:** Hybridization, Hybrid pufferfish, *Takifugu porphyreus*, *Takifugu rubripes*, *Takifugu snyderi*, *Takifugu stictonotus*, Tetrodotoxin

## Abstract

Pufferfish in the genus *Takifugu* accumulate the potent neurotoxin tetrodotoxin (TTX) in specific tissues that vary by species; nevertheless, certain species are consumed as a luxury food in Japan. Therefore, the edible species and tissues of *Takifugu* pufferfish are regulated in Japan. In recent years, unidentifiable individuals with atypical morphological characteristics caught in waters around Japan have been found to be natural interspecific hybrids among *Takifugu* species. Four cases of natural hybridization have been reported; in all cases, hybrids had external characteristics intermediate between those of the parental species. Although the frequency of these hybrids was generally low, it is high in certain parental species combinations. The TTX accumulation in tissues of natural and artificial hybrids was investigated and it was inferred that TTX accumulation in the skin is dominantly inherited. However, information on hybrids’ toxicity in *Takifugu* pufferfish remains insufficient, and further research is required to prevent TTX poisoning associated with them.

## 1. Introduction

Various toxic substances, including tetrodotoxin (TTX), have been found in marine organisms. TTX, which inhibits voltage-gated sodium channels on the nerve/muscle membrane^1^^)^, was first discovered in marine pufferfish belonging to the genus *Takifugu*. Food poisoning due to accidental ingestion of TTX in tissue of pufferfish belonging to the genera *Takifugu* and *Lagocephalus* have occurred in Japan. From 1989 to 2010, this poisoning caused 976 patients and 56 deaths, and it is still occurring frequently^2^^,^^3^^,^^4^^)^. Although TTX was long believed to be contained only in the tetraodontid pufferfish, studies have shown that other marine organisms, such as the goby *Yongeichthys criniger*^5^^)^, gastropod *Babylonia japonica*^6^^)^, starfish *Astropecten polyacanthus*^7^^)^, crab *Atergatis floridus*^8^^)^, and flatworm *Planocera multitentaculata*^9^^)^ also accumulate this toxin, and that several marine bacteria have the ability to produce TTX^10^^,^^11^^,^^12^^)^. Moreover, two pufferfish species (*T. rubripes* and *T. alboplumbeus*) do not accumulate TTX in their bodies when they are reared in environments free from exposure to TTX after hatching^13^^,^^14^^,^^15^^)^. These findings suggest that TTX initially produced by TTX-producing marine bacteria accumulates in pufferfish via the food web^16^^)^.

*Takifugu* species generally accumulate TTX in specific tissues. For example, adult *T. rubripes* and *T*. *xanthopterus* show high toxicity in the liver and ovaries, while the skin, muscle, and testes are hardly toxic^17^^)^. By contrast, other species exhibit high toxicity in the skin, liver, and ovaries, and toxic testes are occasionally found in some species^17^^,^^18^^,^^19^^)^. Based on these reports, the edible species and tissues of *Takifugu* pufferfish are regulated in Japan. Thirteen species of pufferfish in the same genus, including *T*. *rubripes*, are designated as edible. The edible tissues are basically the skin, muscle, and testes, but these vary depending on the species. For example, the edible tissues of the *T*. *rubripes* are these three parts, but that of *T*. *porphyreus* are the muscles and testes as the species accumulates large amounts of TTX in its skin. Therefore, species identification is required for pufferfish to be edible. However, along with identifiable individuals exhibiting typical morphological characteristics, unidentifiable individuals with atypical morphological characteristics are also frequently caught in the waters around Japan. Many of the unidentifiable individuals are natural interspecific hybrids among *Takifugu* species^20^^,^^21^^,^^22^^,^^23^^)^. Moreover, the occurrence of hybrids has increased in recent years; for example, in 2014, hybrids between *T. snyderi* and *T. stictonotus* accounted for nearly 40% of the total catch off the Pacific coast of Eastern Honshu, Japan^22^^)^. The Ministry of Health, Labour and Welfare of Japan states in its notice (Ministry of Health and Welfare, “Hygiene control of pufferfish” in Japanese, 1983) that, for “intermediate species” between two species, the tissues that are designated as edible in both of the two species are likewise permissible for consumption. The term “intermediate species” refers to individuals exhibiting morphological characteristics intermediate between two species, but it does not necessarily indicate interspecific hybrids. If such an intermediate species is interpreted as a hybrid between distinct species, then the edible parts common to the two species are also considered edible. Specifically, in the case of hybrid between *T*. *rubripes* and *T*. *porphyreus*, the muscle and testes are considered edible. However, only one study^23^^)^ has examined this point, so it is difficult to conclude that sufficient knowledge has been obtained. Furthermore, it is difficult to estimate the parental species based on morphological characteristics, making it practically impossible to treat hybrids as food. As mentioned above, hybrid pufferfish have the potential to disrupt the market, so the morphological features, parental combination, and TTX-accumulating tissues must be clarified.

This review describes the occurrence of natural interspecific hybrids between *Takifugu* species in the waters around Japan, including combinations of parental species, external features, and frequency. The TTX-accumulating tissues of natural hybrids and TTX administration experiments in artificial hybrids are also presented.

## 2. Occurrence of Natural Hybrid Pufferfish

**Fig. 1.**
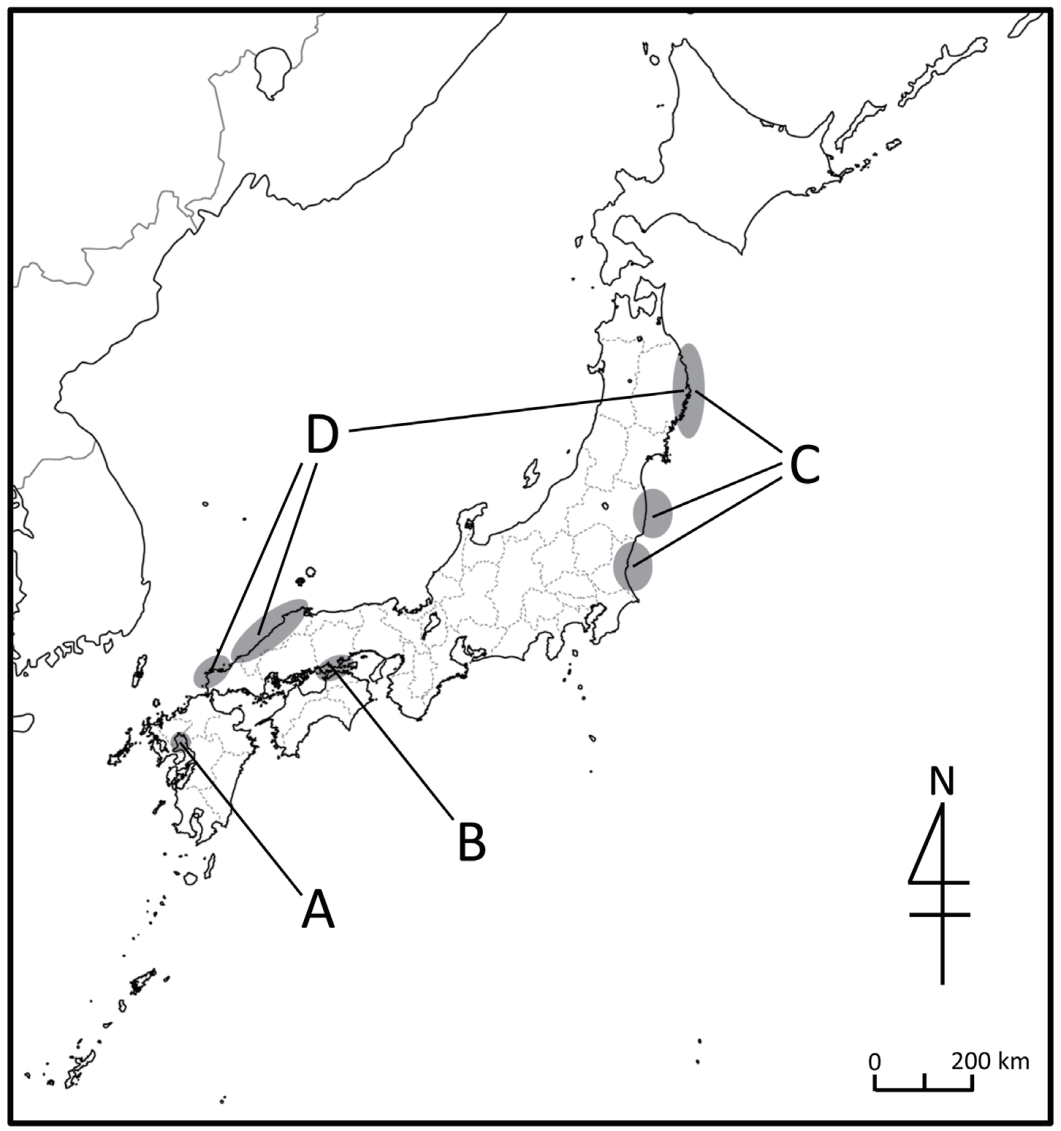
Collection areas for each hybrid pufferfish. The areas A, B, C, and D on the map show where the hybrid between *Takifugu xanthopterus* and *Takifugu vermicularis,* hybrid between *Takifugu vermicularis* and *Takifugu flavipterus*, hybrid between *Takifugu snyderi* and *Takifugu stictonotus*, and hybrid between *Takifugu rubripes* and *Takifugu porphyreus*, respectively, were collected.

Pufferfish of the genus *Takifugu* are closely related species^24^^)^ and their hybrids are occasionally observed in nature. Four papers on natural *Takifugu* hybrids collected from the coastal waters of Japan have been published (**Table 1**, **Fig. 1**). The first report described natural hybrids between *T. xanthopterus* and *T*. *vermicularis* collected in Ariake Bay, western Kyushu^20^^)^. The dorsal and side surfaces of the former are black with several white lines running from the head to the caudal fin, and all fins are bright yellow^25^^)^. The latter have brown dorsal and side surface with irregular white spots, and a dark brown blotch with an irregular white margin behind the pectoral fin^25^^)^. The anal fin is white, whereas the other fins exhibit a mixture of brown and white. The spinules on the body surface are covered in the former, but not in the latter. Six hybrids between *T. xanthopterus* and *T*. *vermicularis* identified by isozyme analysis had black or dark brown dorsal and side surfaces with several partially broken white lines running from the head to the caudal fin; the anal fin was white with light yellow, and the other fins were yellow or dusky yellow; the body surface was covered with spinules that were shorter than those of *T*. *xanthopterus*. These results suggest that these hybrids are morphologically intermediate between *T. xanthopterus* and *T*. *vermicularis*.

**Table 1.** Reports of hybrid pufferfish in the coastal waters of Japan

Parental species	Place of collection	Number of hybrid specimen	Frequency	Reference
*Takifugu xanthopterus*, *Takifugu vermicularis*	Ariake Sea	6	0.4%	Masuda et al., 1991^20^^)^
*Takifugu vermicularis*, *Takifugu flavipterus*	Seto Inland Sea	2	0.05%	Yokogawa and Urayama, 2000^21^^)^
*Takifugu snyderi*, *Takifugu stictonotus*	Eastern Honshu	149	38.5%	Takahashi et al., 2017^22^^)^
*Takifugu rubripes*, *Takifugu porphyreus*	Yamaguchi, Shimane, Iwate Prefecture	10	Less than 1% (estimated)	Tatsuno et al., 2019^23^^)^

The second report on natural hybrids also involved *T*. *vermicularis*. Isozyme analysis determined that two individuals collected in the Seto Inland Sea were hybrids between *T*. *vermicularis* and *T*. *flavipterus*^21^^)^. *Takifugu flavipterus* (formerly *T*. *poecilonotus*) is similar to *T*. *vermicularis* in having brown dorsal and side surfaces with white spots, but differs in having a yellow anal fin, spinules on the body surface, and no dark brown spot bordered by white behind the pectoral fins. As in the first report, the hybrids between *T*. *vermicularis* and *T*. *flavipterus* also had intermediate features, such as weaker spinules than the parental species. These two reports indicate that the external appearance of hybrids are likely to exhibit intermediate features, which have the potential to cause confusion. In addition, little is known about TTX-accumulating tissues in hybrids, which could lead to TTX poisoning if they are sold accidentally. The fact that there are no records of TTX poisoning by these hybrids may be due to their low frequency compared to the parental species, less than 0.5%^20^^,^^21^^)^.

**Fig. 2.**
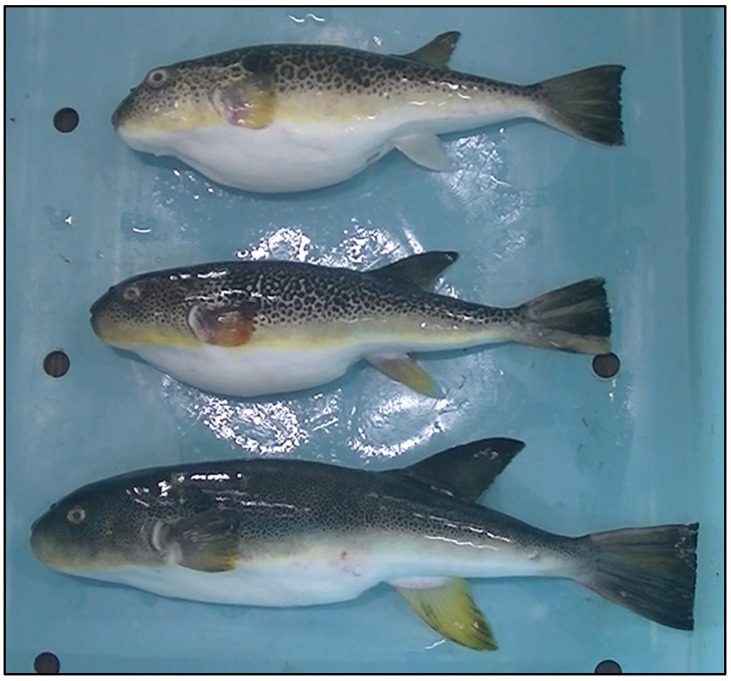
External appearance of *Takifugu snyderi* (upper), *Takifugu stictonotus* (lower), and their hybrid (middle).

In the third report on natural hybrids^22^^)^, the frequency of hybridization was considerably higher. Amplified fragment length polymorphism (AFLP) and mitochondrial DNA (mtDNA) analyses revealed that the parental species of 149 unidentifiable individuals collected from the coastal waters of eastern Honshu were *T*. *snyderi* and *T*. *stictonotus*. Of these, 131 were identified as F_1_ hybrids, of which more than 75% were found to be hybrids between *T*. *stictonotus* females and *T*. *snyderi* males. The estimated frequency of these F_1_ hybrids was 38.5%^22^^)^. Furthermore, this study found 17 first-generation backcrosses to *T*. *snyderi* and one backcross to *T*. *stictonotus*. The parental species are morphologically similar but can be distinguished by differences in anal fin coloration and the presence of spinules on the body surface^26^^)^. *T. snyderi* has a white anal fin and smooth body surface, whereas *T. stictonotus* has a lemon yellow anal fin and its dorsal and ventral surfaces of the body are covered with spinules. Their hybrids have a slightly yellow anal fin and their body surfaces are partly covered with weak spinules (Fig. 2, Table 2). Since the external appearance of these hybrids is intermediate between the parental species, they could be distributed through mishandling. Although the TTX-accumulating tissues of the parental *T*. *snyderi* and *T*. *stictonotus* are the same, there are no published studies of which tissues accumulate TTX in these hybrids. We are currently studying the amount of TTX in the skin, muscle, liver, and gonads of the above hybrids, collected from the coastal waters of eastern Honshu.

**Table 2.** Representative morphological features of *Takifugu snyderi*, *Takifugu stictonotus*, and their hybrid

Species	Color of anal fin	Spinules on body surface
*Takifugu snyderi*	White	No spinules
*Hybrid between* *Takifugu snyderi and Takifugu stictonotus*	Slightly yellow	Weak spinules covered with narrow range compare to *Takifugu stictonotus*
*Takifugu stictonotus*	Lemon yellow	Spinules covered with dorsal and ventral surface

**Fig. 3.**
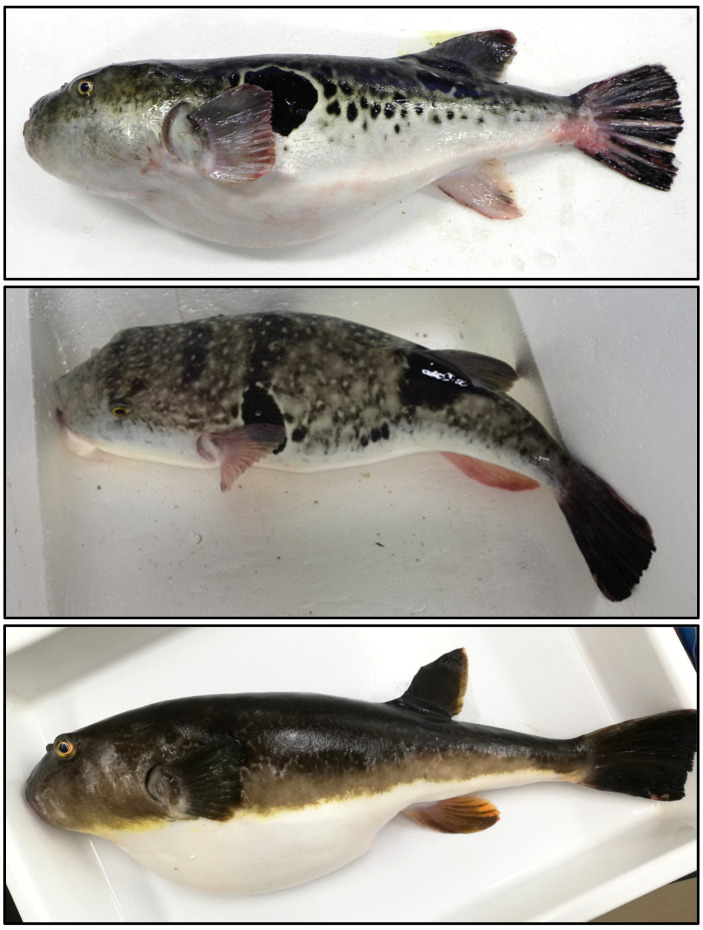
External appearance of *Takifugu rubripes* (upper), *Takifugu porphyreus* (lower), and their hybrid (middle).

The fourth report is on natural hybrids between *T*. *rubripes* and *T*. *porphyreus*^23^^)^. These are commercially important species in Japan, and both are typically sold as live or fresh fish. *T. rubripes* has a black blotch bordered by a white margin behind the pectoral fins, and the body surface is covered with spinules, while *T*. *porphyreus* has a black blotch with no white margin, a yellow line on the body side, and no spinules on the body surface^25^^)^. Using AFLP and mtDNA analyses, 10 unidentified individuals with intermediate morphological features between *T*. *rubripes* and *T*. *porphyreus* (Fig. 3, Table 3) were determined to be hybrids of these species^23^^)^. Hybridization between the two species occurred in both directions; *i.e*., five individuals were F_1_ hybrids between *T. rubripes* females and *T. porphyreus* males and four were the opposite cross. Only one backcross was found in the direction toward *T*. *rubripes*. The estimated frequency of these hybrids is similar to that of the first and second reports. The adults of the two parental species differ in the toxicity of the skin *i.e*., the skin of *T. rubripes* has low toxicity, whereas that of *T. porphyreus* has high toxicity^17^^)^. It is necessary to clarify the toxicity of natural hybrids between two species with different tissue toxicity levels, such as *T. rubripes* and *T. porphyreus*, to understand the inheritance of TTX accumulation. We investigated TTX accumulation in these hybrids and present the results on section 4.

**Table 3.** Representative morphological features of *Takifugu rubripes*, *Takifugu porphyreus*, and their hybrid

Species	A black blotch behind pectoral fin	Spinules on body surface
*Takifugu rubripes*	Black blotch with white margin	Spinules covered with dorsal and ventral surface
*Hybrid between* *Takifugu rubripes and Takifugu porphyreus*	Black blotch with unclear white margin	Weak spinules covered with narrow range compare to *Takifugu rubripes*
*Takifugu porphyreus*	Black blotch with no white magin	No spinules

## 3. Identification of Parental Species of Hybrid Pufferfish

In our previous studies^22^^,^^23^^)^, we identified the parental species of natural hybrid pufferfish using AFLP and mtDNA markers. The AFLP procedure followed the standard method of Vos et al.^27^^)^ with minor modifications, as described by Takahashi et al.^28^^)^. Although AFLP is a somewhat classical technique, it remains a relevant method, as it does not require prior information such as species-specific primer sequences and can thus be readily applied to a wide range of taxa^29^^)^. Moreover, this method enables the detection of numerous polymorphic loci at relatively low cost, allowing discrimination not only of F_1_ hybrids but also of later-generation hybrids using the analytical approaches described below. However, because AFLP relies on detecting polymorphisms in restriction fragments, its applicability is limited for samples with highly degraded DNA. In addition, as AFLP is a dominant marker, the amount of genetic information obtained per locus is relatively limited^30^^,^^31^^)^. Nevertheless, this drawback is generally minor when many markers are fixed for different alleles between the parental species, which is often the case in interspecific comparisons^29^^,^^30^^)^. In recent years, however, AFLP has been largely replaced by reduced-representation genome sequencing approaches, such as restriction-site associated DNA sequencing (RAD-seq) and its derivatives, which generate large numbers of codominant single nucleotide polymorphism (SNP) markers. These next generation sequencing (NGS)-based methods have become the standard for identifying parental species and characterizing hybridization patterns in natural populations^32^^,^^33^^)^.

Parental species identification and hybrid classification of pufferfish samples based on the AFLP data were performed using STRUCTURE^34^^,^^35^^)^ and NewHybrids^36^^)^ in the previous studies^22^^,^^23^^)^. STRUCTURE infers population structure and individual ancestry proportions based on multilocus genotype data. STRUCTURE was run under an admixture model with K = 2 (where K represents the number of genetic clusters, i.e., parental species or populations, assumed in the model), and individuals with ancestry proportions (q) between 0.02 and 0.98 were designated as hybrids^37^^)^. To validate this assumption, it is also advisable to run additional simulations with K ≥ 3 to confirm that no more than two parental species are involved in the hybridization, and to ensure that the inferred genetic structure is not influenced by potential undetected taxa or population substructure. For example, in a study focusing on natural hybrids between *Seriola quinqueradiata* and *S. lalandi*, hybrids between *S. quinqueradiata* and *S. dumerili* were also detected^38^^)^. Such multi-species hybridization events cannot be accurately detected when simulations are performed only under the K = 2 model. NewHybrids assigns individuals to specific hybrid categories (e.g., F_1_, F_2_, and backcrosses) using a Bayesian posterior probability framework. Using NewHybrids, individuals showing nearly equal ancestry proportions from both parental species can be distinguished as either F_1_ or F_2_ hybrids based on their genotype composition, providing further insight into the hybridization stage.

Unlike AFLP markers (and other nuclear DNA markers), mtDNA is maternally inherited and can therefore be used to determine the direction of hybridization, that is, to identify the maternal and paternal species^39^^)^. In our previous studies, we amplified the entire mitochondrial control region (CR) using the primer pair L-Thr-Fugu (5’-TCAGAGCGTCGGTCTTGTAA-3’) and H-12S-Fugu (5’-TCACTGGGWTGCGGATACTT-3’), which were designed based on conserved sites in the tRNA and 12S rRNA genes flanking the CR of *Takifugu* species^24^^)^, under the PCR conditions described by Takahashi et al.^28^^)^. Based on approximately 550 bp of the 5’ half of the CR sequences, which were determined using a capillary DNA sequencer, we reconstructed a phylogeny together with the corresponding sequences of known species and inferred the maternal species of each F_1_ hybrid from its mtDNA lineage. Such mtDNA analysis provided additional information complementary to that obtained from nuclear DNA markers, offering insights into the relationship between the direction of hybridization and the toxicity of hybrids, as well as potential causes underlying interspecific hybridization. It should be noted, however, that mtDNA phylogeny may not always reflect the species or population phylogenies due to introgression via past interspecific hybridization or incomplete lineage sorting^40^^)^; therefore, caution should be exercised when interpreting the direction of hybridization solely from mtDNA-based phylogenetic inferences. For example, *T. stictonotus* mtDNA introgression was detected in two out of 102 genetically pure individuals of *T. snyderi*^22^^)^.

Finally, we introduce a recently developed species/hybrids identification method among all 11 Japanese *Takifugu* species using species-specific SNP markers^41^^)^. These SNP markers were identified through genome-wide RAD-seq analysis of at least eight individuals per species and selected on the basis that one allele was homozygous in all individuals of a given species, whereas the alternative allele was homozygous in all individuals of the other species. The genotypes of these diagnostic SNPs can be determined using TaqMan^®^ genotyping assays on a real-time PCR platform, enabling rapid and reliable identification of species/hybrids even from minute quantities of genomic DNA samples. Furthermore, unlike the AFLP or reduced-representation genome sequencing approaches described above, this method can be applied to highly degraded DNA samples, making it particularly useful for identifying the causative species in food poisoning cases. Although species- and hybrid-diagnostic methods based on species-specific SNP markers have been developed for several fish taxa, most have been limited to two or three potential parental species^42^^,^^43^^)^. Therefore, the present method, which allows discrimination among 11 potential parental *Takifugu* species, represents a remarkable advance. In addition, this approach enables the identification of numerous natural hybrid individuals and facilitates quantitative assessments of their toxicity, thereby contributing to food safety.

## 4. Investigation of TTX-accumulating Tissue Using Natural or Artificial Hybrids

While morphological and genetic studies of natural hybrids among pufferfishes belonging to the genus *Takifugu* have been reported, little is known about their TTX-accumulating tissues. One reason for this may be the difficulty of collecting natural hybrids (except for hybrids between *T*. *snyderi* and *T*. *stictonotus*), as the frequency of hybrids is less than 1%^20^^,^^21^^,^^23^^)^. It is necessary to obtain live or fresh fish to evaluate the amount of TTX in each tissue accurately because TTX migrates between tissues during slow thawing of frozen fish^44^^,^^45^^,^^46^^)^. We collected 10 hybrids between *T. rubripes* and *T. porphyreus*, of which only three (F_1_ hybrids between *T*. *rubripes* females and *T*. *porphyreus* males) were live fish. For TTX quantification by high-performance liquid chromatography with fluorescence detection (HPLC-FLD), the live hybrids were dissected into skin, muscle, liver, and gonads. The four tissues of the remaining seven frozen individuals (two F_1_ hybrids between *T*. *rubripes* females and *T*. *porphyreus* males, four of the opposite cross, and one backcross to *T*. *rubripes*) were dissected out in a half-thawed state to prevent TTX migration. HPLC-FLD detected TTX in the F_1_ and backcross, but in different tissues (**Table 4**). In F_1_ hybrids between *T*. *rubripes* females and *T*. *porphyreus* males, TTX was detected in ovaries, liver, and skin, but not in muscle or testes. Hybrids of the opposite cross also accumulated TTX in the same tissues as F_1_ hybrids between *T*. *rubripes* females and *T*. *porphyreus* males, but differed in that the content level of TTX in the muscles of three individuals was low. The relatively high TTX content in the skin of F_1_ hybrids in both directions suggest a dominant mode of TTX accumulation inheritance in this tissue. The muscle of *T. rubripes* is not known to be toxic, whereas the muscle of 10% of *T*. *porphyreus* had low toxicity (equivalent to 0.4–1.1 µg/g TTX)^17^^)^. Therefore, TTX accumulation in the muscles of hybrids between *T*. *porphyreus* females and *T*. *rubripes* males may be affected by *T*. *porphyreus*, but as the individuals used in this study were all frozen fish, the effects of freezing and thawing cannot be ruled out. In the future, live hybrids should be obtained and verified.

**Table 4.** Toxin amount in each tissues of live specimen of *Takifugu rubripes*, *Takifugu porphyreus*, and their hybrid

Species	Condition	Total number of specimens	TTX content (µg/g)				
			Skin	Muscle	Liver	Ovaries	Testes
F1 hybrid between *Takifugu rubripes* females and *Takifugu porphyreus* males	Live specimen	3	<0.1-17.4	<0.1	3.2-171.6	8.4-181.6	<0.1
F1 hybrid between *Takifugu rubripes* females and *Takifugu porphyreus* males	Frozen specimen	2	<0.1-0.2	<0.1	<0.1-1.4	10.1	<0.1
F1 hybrid between *Takifugu porphyreus* females and *Takifugu rubripes* males	Frozen specimen	4	0.8-8.2	<0.1-1.1	0.2-580.8	68.1-376.2	-
*Backcross toward* *Takifugu rubripes*	Frozen specimen	1	0.5	<0.1	9.8	-	<0.1
*Takifugu rubripes* ^17^^)^	Live or fresh specimen	43	<0.1-0.4^※^	<0.1^※^	<0.1-44.0^※^	<0.1-110.0^※^	<0.1^※^
*Takifugu porphyreus* ^17^^)^	Live or fresh specimen	53	<0.2-44.0^※^	<0.2-1.1^※^	<0.2-220.0^※^	0.4-440.0^※^	<0.2-1.1^※^

※ The value converted from mouse unit to µg (TTX equivalent)

Studies have examined the TTX-accumulating tissues in both natural and artificial *Takifugu* hybrids. As mentioned above, pufferfish of the genus *Takifugu* do not have TTX if reared on a TTX-free diet from hatching. The accumulation of TTX in each tissue can be quantified by administering TTX to such artificial hybrids, as described in two papers. In experiments using artificial hybrids between *T*. *rubripes* and *T*. *alboplumbeus* (formerly known as *T*. *niphobles*), TTX was injected into the muscles to investigate TTX accumulation in each tissue^47^^)^. The latter, like *T*. *porphyreus*, has been reported to accumulate large amounts of TTX in the skin^19^^)^. Liquid chromatography–mass spectrometry analysis revealed that the administered TTX was first taken up in the liver and then transferred to the skin. No TTX was detected in the muscles or testes. Similar findings were reported in artificial hybrids between *T. rubripes* females and *T. porphyreus* males administered TTX orally or intramuscularly^48^^)^. These observations of artificial hybrids suggest that TTX accumulation in the skin is inherited dominantly. However, further research should assess this hypothesis because the main TTX-accumulating tissue in *T*. *rubripes* changes depending on the growth stage^49^^)^. In *T*. *rubripes*, TTX is transferred mainly to the skin in 6-month-old individuals, while 15-month-old individuals accumulate TTX mainly in the liver and less in the skin^49^^)^. Furthermore, 3-year-old *T*. *rubripes* accumulated no TTX in the skin, and the liver or ovaries were the main tissues accumulating TTX^50^^)^. The above two studies of artificial hybrids used 8- to 10-month-old individuals. Based on these findings, future experiments should administer TTX to artificial hybrids at each growth and maturation stage to investigate the transition of the main TTX-accumulating tissues.

## 5. Conclusion

Natural hybrids of the genus *Takifugu* occur in the waters around Japan. The external features of hybrids, such as color patterns and spinules, may confuse the fish market because they are intermediate between the parental species. Although most natural hybrids occur at low frequency, a significant number of individuals may occur, such as hybrids between the *T*. *snyderi* and *T*. *stictonotus*. Therefore, it is necessary to collect information on the occurrence of natural hybrids and to disseminate this information to the fish market. Among the natural hybrids of *Takifugu* species, the TTX-accumulating tissues have only been examined in the hybrid between *T*. *rubripes* and *T*. *porphyreus*. These parental species differ in the accumulation of TTX in the skin, with *T*. *rubripes* accumulating little TTX in skin, while *T*. *porphyreus* accumulates large amounts. The hybrids accumulated large amounts of TTX in the skin, suggesting that TTX accumulation in the skin was dominantly inherited. In the future, we should investigate whether similar phenomena occur in hybrids of other combinations of parental species and elucidate the molecular mechanisms responsible for this.
